# THE COVID-19 PANDEMIC MAY HAVE EXACERBATED NURSE STAFFING DISPARITIES IN NURSING FACILITIES

**DOI:** 10.1093/geroni/igac059.1317

**Published:** 2022-12-20

**Authors:** Kristie Porter, Angela Gasdaska, Marie Squillace, Judith Dey, Iara Oliviera, Zhanlian Feng, Micah Segelman

**Affiliations:** RTI International, Durham, North Carolina, United States; RTI International, Research Triangle Park, North Carolina, United States; ASPE, Washington, District of Columbia, United States; Office of the Assistant Secretary for Planning and Evaluation, Washington, District of Columbia, United States; Office of the Assistant Secretary for Planning and Evaluation, Washington, District of Columbia, United States; RTI International, Research Triangle Park, North Carolina, United States; Research Triangle Institute, Washington, District of Columbia, United States

## Abstract

Nursing facilities (NFs) have historically struggled to maintain adequate nurse staffing. We used PBJ data linked with other publicly available sources and conducted stakeholder interviews to understand nurse staffing between 2019 and 2020. We found large declines in the population of NF residents and in staffing hours. Measured in hours per resident day (HPRD) to account for the size of the NF resident population, there were slight increases in staffing. Staffing was nonetheless a major challenge for NFs because they required increased staffing due to the impact of the pandemic. NFs in higher quartiles of percentage of minority residents lost nurse staffing HPRD relative to NFs in the lowest quartile of minority residents. Stakeholders explained that NFs with more minority residents were likely to employ staff who live in more vulnerable communities with a greater concentration of minorities, who were more impacted by COVID.

